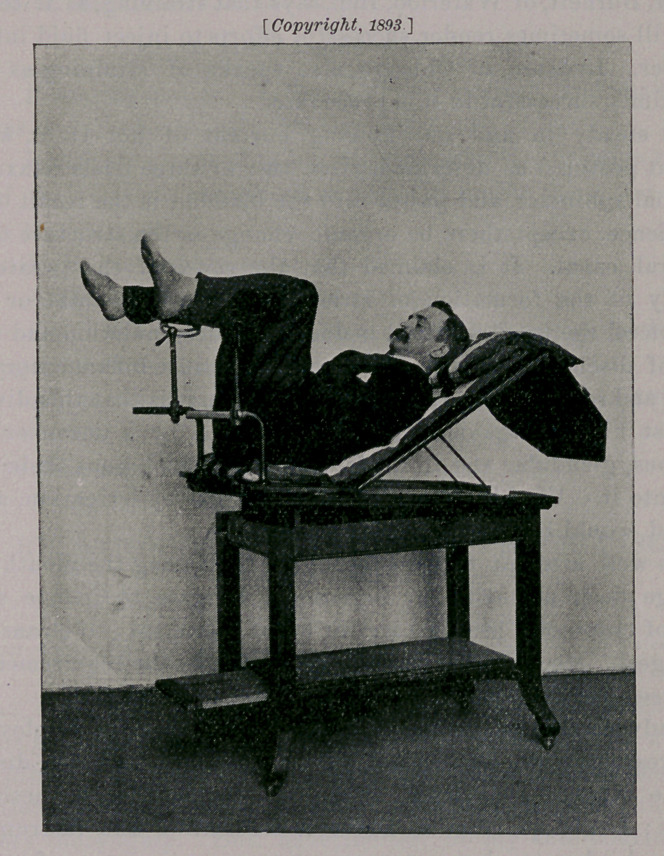# Irrigation of the Urethra and Bladder by Posture and Continuous Current1Read at the regular quarterly meeting of the Lake Erie Medical Society, July 15, 1892.

**Published:** 1893-03

**Authors:** B. H. Daggett

**Affiliations:** Buffalo, N. Y.; 258 Franklin Street


### Buffalo Medical and Surgical Journal

Vol. XXXII. MARCH, 1893. No. 8.

### Original Communications.

### IRRIGATION OF THE URETHRA AND BLADDER BY POSTURE AND CONTINUOUS CURRENT.[1]

By B. H. DAGGETT, M. D., Buffalo, N. Y.

The management of urethral disease is a reproach to the profession. The fact that numberless and divers formulæ for use in this malady burden our text-books and encumber our journals, indicates that the profession is at sea, that our methods are uncertain, and our treatment is empirical. The bacteriologist is busy in discovering pathological germs and the therapeutist is baffled in his combat with them. Neisser discovered the gonococcus and alleged that this is the cause of specific urethritis. The therapeutist forthwith applied germicidal remedies, and a new set of formulæ were launched upon the medical sea of uncertainty.

The germ theory of specific urethritis has not been universally accepted, and surely has not begotten any more successful method of treatment than formerly prevailed. My experience and observation teach me that germicidal, or any other remedies which aggravate the existing irritation, do harm ; for germs revel in the products of inflammatory conditions.

Otis says that corrosive sublimate is often irritating, even when one part to 20,000 is used. Gouley says not to give injections during the acute stage of urethritis. Meddlesome and inappropriate instrumentation have done irreparable harm to the genito-urinary apparatus. The urethral canal is a collapsing tube, curved in its course, flattened transversely or longitudinally in the different divisions of its route ; it is supported by cellular, expansible tissue, and lined with a delicate, sensitive, folding membrane ; and is part and parcel of two of the most functionally active organs of the body.

1. Read at the regular quarterly meeting of the Lake Erie Medical Society, July 15, 1892.

Yet this delicate, complicated structure is incised, divulsed, punctured, corroded by chemicals and electricity; eroded by injudicious use of sounds and catheters as freely as though it were the commonest of structures. Instrumentation of the urethral tract is not unsurgical or unnecessary, but it is to be avoided during the existence of acute urethritis and its sequelæ; orchitis, epididymitis, cystitis, pyelitis and vesiculitis.

While attending the surgical ward of the Charity Hospital a few years ago, there came under my care a patient suffering from chronic gonorrhea and cystitis, who could not speak English sufficient to give a history of his case. The sound indicated narrowing of the bulbous division of the urethral canal, which was duly incised. The patient was not benefited by this treatment; in fact, his condition was aggravated by an attack of urethral fever. The disease extended to the kidneys, and after a few weeks, like Asa of Sacred Writ, he slept with his fathers. There was no complaint, no investigation, no coroner. This lesson was not forgotten, and is now fully emphasized by the genito-urinary surgeons, who warn us of the danger of internal urethrotomy beyond the external sphincter. The urethral canal should not be instrumentally invaded during the course of active inflammation or during the existence of any of its concomitants. Should retention occur, it is safer to alleviate by aspiration; or if the retention is due to some pathological condition of the genito-urinary apparatus, it is better to make a false urethra by puncturing the perineum about three-fourths of an inch above the anal verge, pass the instrument directly to and through the apex of the prostate, when a catheter may be introduced and free drainage maintained. This procedure is fully described by Reginald Harrison. The bladder being drained in this way the offending constrictor and sphincter muscles become quiescent and certain morbid conditions are thereby relieved, as certain rectal diseases are healed by stretching the sphincter ani. Instrumentation aggravates engorged and irritable urethræ precisely as it does rhinital hypertrophy. This is readily demonstrated by probing nares, narrowed by spongy, hypertrophied tissue, or the granular membrane of middle ear catarrh, which causes puffiness, secretion, and pain.

It is well known that a small pile tumor or a fissure within the grasp of the sphincter ani muscle will cause severe pain; and that fissure and rectal engorgement are readily healed as well as relief given to pain by muscle rest. While the posterior urethra is not so highly endowed with sensation as to give warning by acute pain, yet one can understand by analogy how rest of the vesical structures may exercise a curative influence. There is another complication which may occur when there are lesions in the grasp of the muscles of the urethral tract; namely, puddling and retention of urine, its admixture with blood and serum, which decompose and form toxic agents, causing local irritation and systemic infection, or urethral fever. For this reason internal urethrotomy of the deep portion of the canal posterior to the external sphincter is superseded by dilatation, or the external operation, which avoids this difficulty by providing free drainage.

Normal flowing urine is not baneful, hence operative procedures which provide for drainage are not followed by the disturbances which frequently pursue lesions of the deep urethra.

Several years ago, while endeavoring to blow a powder into the urethra for relief of a chronic urethritis, by pressing the blunt nozzle of a powder blower into the pouting lips of the urethral meatus, my patient expressed an urgent desire to pass water; attempting to urinate, he passed wind and no water. I repeated this procedure several times with success. This disclosure suggested the feasibility of irrigating the deep urethra and bladder without encroaching instrumentally upon the urethral tract. I endeavored to inject water in a similar way and failed. I found by accident a Kiefer nozzle, which was designed for irrigating the anterior urethra. I had no hope of reaching the deep urethra or bladder.

FIG. 1. Kiefer Nozzle. FIG. 2. Daggett Cannula.

My patients were induced to irrigate the urethra, using water as hot as it could be borne; to place themselves in a bath tub with the back resting upon the incline at the head of the tub, and flex the lower extremities. They all expressed themselves as being much relieved by the treatment, and as it produced prompt results they were faithful in carrying out the instructions. Nearly all the cases reported that the water entered the bladder. One patient reported that he filled the bladder a dozen times the first séance.

Tieman and Company have constructed, at my request, a double cannula, Y-shaped, with a nozzle about three-fourths of an inch long. It is enclosed in a thin, inflatable, rubber sac, similar to the Gihon urethral tampon. This is introduced into the urethra so that as the bag is blown up it fills the navicular fossa, dilates the anterior urethra, and holds the cannula in place; the cannula may also be held by a modified Mitchell hood. A fountain syringe is filled with hot water, and placed from two to four feet higher than the pelvis, first being connected with the inlet tube of the cannula. The inlet is a trifle larger than the outlet. The instrument should be made of hard rubber, or some material that is a poor conductor of heat. Irrigation is a coaxing process and cannot be successfully forced. The constrictor muscles resent the impact of an injection as well as force of any kind. A tube is attached to the cannular outlet to carry off the waste.

The cannula is held by placing the forefinger in its bifurcation, and the gland is grasped behind its corona by the thumb and other fingers; the stop cock is turned and the water is felt working its way along the canal and passing imperceptibly into the bladder, causing a desire to urinate after an accumulation of sufficient bulk.

Water sufficiently hot to cause smarting of the skin is borne with a feeling of relief and comfort. If the disease is confined to that portion of the urethra anterior to the external sphincter, the irrigation is to be done in the upright position, but if the disease has extended beyond this barrier and involves the deep urethra or bladder, or both, the irrigation should be carried to these parts. The only obstacle to be overcome is the mixed muscular structure of the external sphincter; beyond this the way is clear, for the internal sphincter relaxes from the presence of fluid in the deep urethra. To overcome this obstacle it is essential to suitably posture the patient. An anterior urethritis may extend beyond the external sphincter in from one to three weeks' time.

To successfully carry out irrigation of the deep urethra and bladder, the patient should be placed upon his back, in the reclining position, for the purpose of securing general relaxation and giving a downward dip to the horizontal or fixed portion of the urethral canal. The shoulders should be raised, thus flexing the upper portion of the body upon the pelvis, which is also slightly raised, the thighs are flexed and legs are supported by crutches to obviate tension. These flexures are in imitation of nature's method of relaxing the pelvic floor and its associated structures. It is well to bear in mind that the neck of the bladder is one and one-quarter inches behind and a little below the middle of the symphysis in the upright posture, and in the horizontal it is one-half inch lower.

This posture also gives the surgeon a chance to aid the passage of the sound or catheter when deeply obstructed, by passing the

[*Copyright, 1893.*]

finger into the rectum and guiding the instrument through the external sphincter and membranous urethra. Nature's method of relaxing these structures is indicated by the fact that all animals squat and hump the back when evacuating the pelvo-abdominal emunctories. Thus they relax the muscles of the pelvic floor as well as the sphincters, which have a direct connection by interchanging muscular fibers and are intimately associated by nerve distribution.

The penis should be drawn tense, in order to straighten the canal and unfold the mucous membrane as much as possible. It will be observed that as the horizontal posture lowers the neck of the bladder that traction will aid in making comparatively straight the urethral way. During the process of irrigation the attention of the patient should be diverted.

Dr. Burnett, of Waterloo, Ind., says that straining, as if to urinate, will sometimes render successful efforts to inject fluid into the bladder. Lydston, of Chicago, also speaks of straining as being sometimes successful in this procedure.

A steady in and out flowing current of hot water, with a patient postured as described, after two or three trials relaxes the external sphincter and passes into the bladder, is the result of my experience, except there be organic change in the structure of the urethral canal. It is claimed that the cause of the greater frequency of the formation of stricture in the horizontal or fixed portion of the urethral canal is due to the accumulation and retention of discharges, which decompose and cause inflammatory processes and resulting plastic exudate. Certain it is that this division is most highly organized and is penetrated by a dozen or more patulous, glandular and follicular openings which pour their secretion into it. Washing away these discharges by irrigation, as described, would be preventive of stricture.

As well attempt to treat laryngitis by rinsing the mouth as to manage deep urethritis by injection. Irrigate to induce a due state of chronicity preparatory for other treatment; to cleanse and decongest. Medicate the irrigant according to the requirements of the case—use dehydrates, decongestives, avoid irritants.

I advocate irrigation as one of the measures to be employed in the treatment of complicated, stubborn, as well as acute, diseases of the urinary apparatus. I do not discredit other necessary means; I only question meddlesome medication and instrumentation, and advocate hot water irrigation as a preventive of complications and a preparative for other measures, as well as a remedial agent.

The prostate muscle is the male womb; following up the analogy we might learn a valuable lesson from our confrerés, the gynecologists, who have adopted the use of hot water irrigation, ceased cauterizing erosions of the uterus, and consigned the uterine sound—that inciter of pelvic woe and menace to the legacies of love—to "innocuous desuetude." Yet it is a curious fact that the uterine sound is one of the best selling instruments in the surgical market. Several years ago Potter, of Buffalo, sounded the alarm in The Rise and Progress of the Uterine Sound, the second edition notes its Fall. The urethral sound is a useful, essential instrument, when guided by skill, judgment, and experience. It should not be passed with the patient in the upright position, or without at least flexure of the thighs.

RULES FOR PASSING THE SOUND.

1. Place the patient in the reclining posture, and make the flexures described—at least flex the thighs.

2. Lubricate the urethra by injecting alboline, or some refined oil; if the oil is not cocainized and it is desirable to use cocaine, it should be first applied.

3. Make the urethra tense by traction.

4. Avoid force; go slow; be patient.

5. Guide the instrument if obstructed in the deep urethra, by the finger introduced into the rectum.

6. All instruments are to be made surgically clean and perfectly smooth.

258 Franklin Street.

## Figures and Tables

**Fig. 1. f1:**
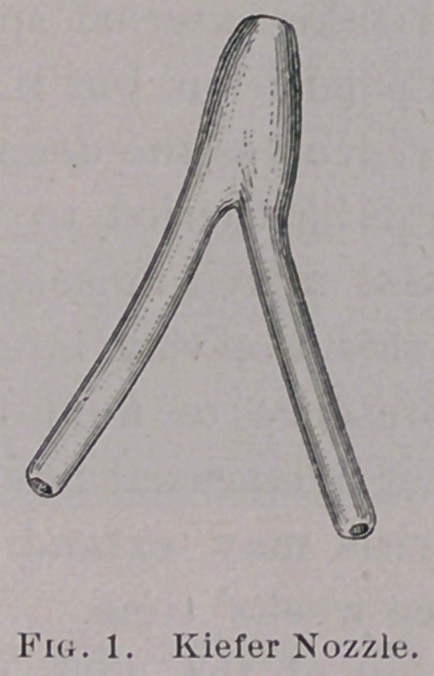


**Fig. 2. f2:**
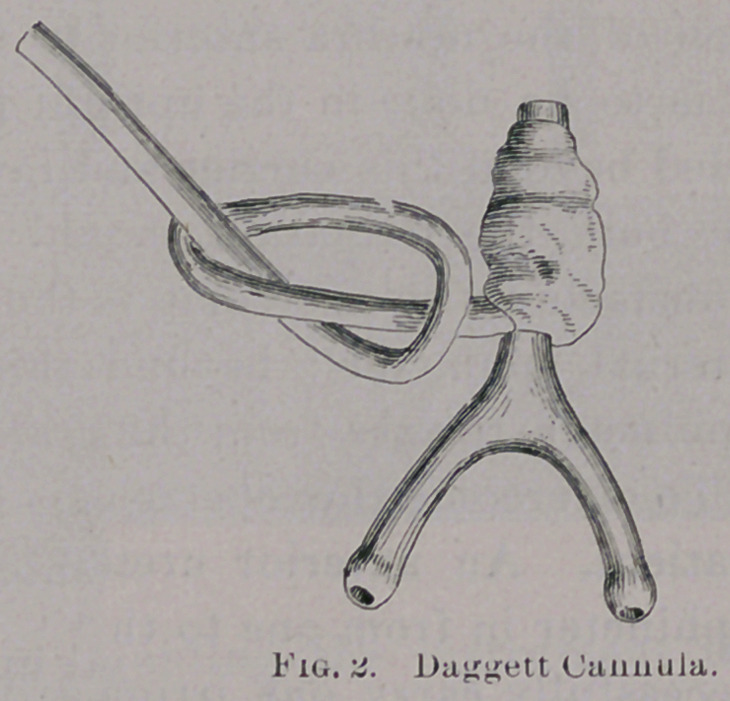


**Figure f3:**